# Resveratrol as a naturally occurring inflammasome modulator: Implications for health and disease

**DOI:** 10.22038/ijbms.2025.87366.18879

**Published:** 2025

**Authors:** Mahboobeh Ghasemzadeh Rahbardar, Amirhossein Sahebkar

**Affiliations:** 1 Clinical Research Development Unit, Shahid Hasheminejad Hospital, Mashhad University of Medical Sciences, Mashhad, Iran; 2 Biotechnology Research Center, Pharmaceutical Technology Institute, Mashhad University of Medical Sciences, Mashhad, Iran; 3 Centre for Research Impact & Outcome, Chitkara College of Pharmacy, Chitkara University, Rajpura 140401, Punjab, India; 4 Applied Biomedical Research Center, Mashhad University of Medical Sciences, Mashhad, Iran

**Keywords:** Cardiovascular diseases, Cytokines, Digestive system diseases, Inflammation, Interleukins, Nervous system diseases, Oxidative stress, Phytochemical

## Abstract

Inflammation plays a crucial role in the pathophysiology of diverse diseases, ranging from chronic disorders to acute inflammatory responses. Inflammasome, a multiprotein complex that controls the activation of pro-inflammatory cytokines and initiates immunological responses, has emerged as a critical regulator of inflammation. Resveratrol, a polyphenolic substance present in grapes, berries, and peanuts, has drawn significant attention due to its potential health benefits. The purpose of this review article is to investigate the role of resveratrol as a naturally occurring inflammasome modulator as well as its implications for health and illness. The present review provides a comprehensive summary of the current evidence on the effects of resveratrol on inflammasome activation and regulation, highlighting *in vitro *and *in vivo* studies investigating the effect of resveratrol on different inflammasome pathways. Furthermore, the underlying mechanisms through which resveratrol affects the nervous system and cardiovascular, respiratory, renal, digestive, and reproductive system disorders are discussed. The extant evidence suggests that various mechanisms are involved in the therapeutic effects of resveratrol, including inhibiting NLRP3 inflammasome activation and subsequent release of IL-1β, IL-18, and TNF-α, reducing inflammation and oxidative stress, activating the Nrf2 pathway, and preventing NF-κB signaling. However, further research is required to determine the therapeutic significance of resveratrol in the prevention and treatment of inflammatory disorders, particularly in clinical practice, as well as to completely unravel the specific molecular processes behind these properties. In conclusion, resveratrol is a promising naturally occurring inflammasome modulator that has the potential to reduce inflammation and related diseases.

## Introduction

The physiological process of inflammation has been present throughout biological evolution and is characterized by the activation of immunological and non-immune cells. Its primary function is to protect the host organism from various threats, such as infections, toxins, bacteria, and viruses. This is achieved by efficiently eliminating harmful pathogens and promoting the healing and regeneration of damaged tissues (1, 2). The typical inflammatory response is distinguished by a time-limited increase in inflammatory processes that occurs in the presence of a threat and subsides after the threat is removed (2-4). Nonetheless, various social, psychological, environmental, and biological variables have been linked to the inability of acute inflammation to resolve. As a result, these variables lead to the development of a chronic state of low-grade, non-infectious systemic chronic inflammation. This state is characterized by the activation of immune components that often differ from those involved in an acute immune response (3, 5). The transition from a short-term to a long-term inflammatory response can impair immunological tolerance, causing major changes in all tissues and organs, as well as normal cellular functioning. This, in turn, increases vulnerability to several non-communicable illnesses in people of various ages (2, 4). Furthermore, systemic chronic inflammation might compromise immune system function, increasing sensitivity to infections and malignancies and a reduced response to immunizations. Furthermore, systemic chronic inflammation during pregnancy and childhood might have serious developmental implications, increasing the lifelong risk of non-communicable illnesses (6). 

Various recognition mechanisms have co-evolved over time to distinguish between the preservation of internal balance (homeostasis) and threats posed to the host. Certain receptors have evolved to recognize particular common patterns known as pathogen-associated molecular patterns (PAMPs) among these systems. These patterns are found mostly in microbes, allowing for the exact identification of infections even in tissues where they are normally absent. Furthermore, several receptors are crucial for identifying signals originating from the host, known as damage-associated molecular patterns (DAMPs). When tissue homeostasis is interrupted by either microbial or non-microbial factors, these DAMPs are produced, allowing for a more comprehensive identification of stressed tissues (7, 8). 

A group of protein complexes known as inflammasomes may recognize a variety of factors that might cause inflammation. Both PAMPs and DAMPs are included in these stimuli. Interleukin (IL)-1β and IL-18 are two essential pro-inflammatory cytokines that are produced under the control of inflammasomes (7, 9). In general, the inflammasome is characterized by the presence of a sensor protein, which acts as a pattern recognition receptor (PRR), and its primary role is to stimulate the inflammatory response by boosting the maturation and production of pro-inflammatory cytokines. The importance of inflammasomes lies in their capacity to fine-tune immune responses and maintain immunological homeostasis. It is essential for host defense against infections as well as for recognizing and clearing damaged or malfunctioning cells (10). However, inflammasome activation that is dysregulated or prolonged can cause chronic inflammation and contribute to the development of a variety of inflammatory diseases, including autoimmune disorders, metabolic diseases, and neurodegenerative complications (11). Understanding the underlying mechanisms and regulation of the inflammasome has important implications for developing therapeutic strategies to modulate inflammation and treat associated diseases. 

Medicinal plants and their phytoconstituents have long been used and have played an important role in the treatment of a variety of illnesses, including inflammatory disorders (12-14). Furthermore, active constituents derived from herbs are being increasingly acknowledged as promising sources for the discovery of innovative therapeutic candidates. 

A bioactive polyphenolic substance called resveratrol (3,5,4′-trihydroxystilbene) is found naturally in many plants, including blueberries, mulberries, grapes, red rice, apples, black olives, red fruits, capers, pistachios, and peanuts (15-18). Plants produce resveratrol as a defense mechanism against environmental stress and potential pathogenic invasions (16). Previous investigations have shown its hepatoprotective (19), neuroprotective (20), cardioprotective (21), anti-cancer (16), anti-aging (22), anti-diabetic (23), antimicrobial (24), and wound-healing (25) properties, which could be attributed to its anti-oxidant (26), anti-inflammatory, and anti-apoptotic (27) effects. The anti-inflammatory effect of resveratrol has been indicated in numerous studies. For instance, it effectively prevented lymphocytes from creating IL-2 and interferon-gamma (IFN-γ), as well as macrophages from releasing tumor necrosis factor-alpha (TNF-α) or IL-12 (28). Besides, resveratrol reduced the production of TNF-α, IL-1α, and IL-6 and down-regulated both messenger ribonucleic acid (mRNA) expression and protein release of IL-17 (29). Moreover, some other investigations revealed its inhibitory effect on inflammasomes (30-32). 

This review article aims to provide a comprehensive overview of the role of resveratrol as a naturally occurring inflammasome modulator. We also sought to explore the potential mechanisms underlying the anti-inflammatory properties of this compound and its therapeutic implications for various health conditions, including inflammatory disorders, autoimmune diseases, metabolic disorders, and neurodegenerative complications.

## Methods

This review was directed at gathering information from scientific databases, such as PubMed, Scopus, and Web of Science, covering the period from their inception to May 2025. The search focused on evaluating the physio-pharmacological properties of resveratrol, using keywords such as “inflammasome”, “inflammation”, “anti-inflammatory”, “cytokines”, “resveratrol”, “neuro-inflammation”, “neuro-protective”, “nervous system”, “cardiovascular system”, “cardioprotective”, “respiratory system”, **“**reproductive system**”,** “lung”, “renal system”, “urinary system”, “kidney”, “digestive system”, “lung”, and “intestine.” 

### Inclusion criteria

Studies were included in this review if they met the following criteria:

- Published in peer-reviewed journals.

- Investigated the effects of resveratrol on inflammasome activation or related inflammatory pathways.

- Included *in vitro* or *in vivo* studies.

- Available in English.

### Exclusion criteria

Studies were excluded based on the following criteria:

- Non-peer-reviewed articles, such as editorials, commentaries, and opinion pieces.

- Studies that did not specifically address the role of resveratrol in modulating inflammasomes or inflammation.

- Animal studies that did not provide relevant data on the mechanisms of action of resveratrol.

### Inflammasome activation and regulation

Inflammasomes are protein complexes that become activated in order to initiate an immune response against cellular damage and pathogens. The inflammasome, a key signaling pathway in the innate immune system, is made up of many proteins that activate caspase-1 (33, 34). They are composed of several proteins, including a sensor molecule (such as nucleotide-binding oligomerization domain-like receptor family pyrin domain-containing (NLRP)1, NLRP3, absent in melanoma 2 (AIM2), or Pyrin), an adaptor molecule (apoptosis-associated speck-like protein containing a CARD domain (ASC)), and an effector molecule (caspase-1) (35). Scientific literature has described a number of inflammasome complexes, including NLRP1, NLRP3, NLR family CARD domain-containing protein 4 (NLRC4), and NLRP6. The intracellular Nod-like receptor (NLR) family includes several complexes, which are classed as PRRs (33). Inflammasome activation leads to the processing and secretion of the pro-inflammatory cytokines IL-1β and IL-18, as well as the induction of pyroptosis, a form of inflammatory cell death (35).

Activation of the inflammasome typically occurs in two steps: priming and activation. During the priming step, PRRs recognize PAMPs or DAMPs on microbial pathogens or damaged host cells, respectively (36). This recognition triggers the production of pro-inflammatory signals, such as nuclear factor-kappa B (NF-κB)-dependent cytokines (TNF-α and IL-1α), which act as “priming signals” to up-regulate the expression of inflammasome components (37, 38).

The activation of the inflammasome complex itself is the second stage. This happens when the sensor molecule detects certain danger signals, such as chemicals produced by pathogens or danger signals originating from hosts that are released during cellular stress or damage. The precise process of activation varies depending on the sensor molecule, but it frequently involves conformational changes and oligomerization of the inflammasome components, which leads to caspase-1 recruitment and activation. After that, activated caspase-1 cleaves the pro-forms of IL-1β and IL-18 into their active forms, which are then released from the cell (39-41).

Inflammasome activation and subsequent cytokine release are tightly regulated to prevent excessive or prolonged inflammation, which can lead to tissue damage and chronic inflammatory diseases (42). Inflammasome activity is regulated by a number of mechanisms, including: 1. Various substances, such as cytokines, enzymes, and regulatory proteins, can inhibit inflammasome activity. Inflammasome activity and cytokine production can be suppressed by IL-10 and transforming growth factor-beta (TGF-β). Caspase-8, NLRP12, and cylindromatosis lysine 63 deubiquitinase (CYLD) are other negative regulators that can block inflammasome signaling at various stages (43-45). 2. Autophagy, a cellular process that involves the degradation and recycling of cellular components, has the potential to control inflammasome activity. Autophagy can degrade inflammasome components, inhibiting their aggregation and activation. Autophagy may also remove damaged mitochondria, which can be a source of inflammasome-activating signals (46). 3. Phosphorylation, ubiquitination, and other post-translational changes can all influence inflammasome function. Phosphorylation of inflammasome components, for example, can either promote or prevent their activation. Ubiquitination can degrade inflammasome components or change their location and function (47). 4. Pathogens have evolved a variety of mechanisms to avoid or limit the activation of inflammasomes. Some bacteria, for example, can release effector chemicals that directly target inflammasome components or disrupt downstream signaling cascades (48).

Understanding the underlying mechanisms and regulation of inflammasome activation is critical for developing therapeutic methodologies to regulate inflammation in a variety of illnesses. Inflammasome-related pathways are being investigated in an attempt to uncover possible targets for managing inflammatory disorders such as auto-inflammatory diseases, metabolic disorders, and neurodegenerative diseases.

### Resveratrol role as an inflammasome modulator

***Nervous system***

Inflammation plays a significant role in the pathogenesis of various nervous system disorders, including pain, Alzheimer’s disease (49, 50), neuropathic pain (51, 52), Parkinson’s disease (53), depression (54), and neurotoxicity (55, 56). There are several causes of chronic inflammation in the central nervous system (CNS), including infections (57), autoimmune responses (58), and the accumulation of misfolded proteins (59). Inflammatory processes in the CNS involve the activation of immune cells, such as microglia and astrocytes (60), which release pro-inflammatory cytokines (61). The inflammasome is a crucial component of the innate immune system, responsible for initiating the inflammatory response to various stimuli (62). According to a growing body of research, inflammasomes have been implicated in the development of a variety of nervous system disorders (63, 64). Inflammasome activation dysregulation has been linked to neuro-inflammation (65), neuronal injury (66), and neurodegenerative disorders such as Alzheimer’s (67), Parkinson’s (68), and multiple sclerosis (69). Inflammasome activation can cause an excessive release of pro-inflammatory cytokines, notably IL-1β, which can result in oxidative stress, chronic inflammation, and the impairment of neuronal function and survival (70-72). 

This association between inflammasome activation and nervous system disorders has prompted investigations into potential therapeutic strategies that can modulate the inflammasome pathway. One such candidate is resveratrol.

*In vitro*

In a murine microglial cell model of inflammatory injury induced by amyloid-β, it was observed that resveratrol exhibited significant inhibition of amyloid-β-induced proliferation and activation of BV-2 cells. Furthermore, resveratrol effectively reduced the release of pro-inflammatory cytokines IL-6 and TNF-α. The study also found that resveratrol suppressed the overexpression of cleaved caspase-1 and IL-1β and attenuated amyloid-β-induced degradation of inhibitor of kappa B alpha (IkBα) and phosphorylation of NF-κB. Additionally, Western blot analysis demonstrated that Aβ up-regulated the Thioredoxin interacting protein (TXNIP)/Thioredoxin (TRX)/NLRP3 pathway, while treatment with resveratrol inhibited this pathway (73). Treating SH-SY5Y cells exposed to oxygen-glucose deprivation remarkably increased cell viability, mRNA levels of nuclear factor erythroid 2-related factor 2 (Nrf2), catalase (CAT), and heme oxygenase-1 (HO-1), enhanced the activity of Nrf2, superoxide dismutase (SOD), and glutathione peroxidase (GPx), as well as the amounts of glutathione (GSH). Furthermore, it attenuated TNF-α, IL-1β, and IL-18 levels and lowered mRNA levels of inhibitor of nuclear factor Kappa-B kinase subunit alpha (IKKα), IKKβ, p65, NLRP3, and caspase-1. It also decreased the activity of caspase-1 (20). The potential of resveratrol to provide protection against chronic neuro-inflammation was demonstrated by a study that focused on metabolic changes in the absence of external stresses. Resveratrol could attenuate inflammasome activity, cellular metabolism, and mitochondrial activity while increasing insulin-like growth factor 1 release as well as glucose uptake in the HMC3 human microglia cell line (74). Another *in vitro* study aimed to investigate the regulatory mechanisms underlying the anti-inflammatory properties of resveratrol in the N9 microglial cell line. The findings revealed that resveratrol effectively suppressed the activation of the NLRP3 inflammasome induced by lipopolysaccharide and adenosine triphosphate (ATP), thereby defending microglial cells against oxidative stress, pro-inflammatory cytokine production, and pyroptotic cell death associated with inflammasome activation. Moreover, resveratrol inhibited the signaling of NF-κB and activated the adenosine monophosphate-activated protein kinase (AMPK)/Sirtuin 1 (Sirt1) pathways. Additionally, the results demonstrate that resveratrol down-regulated the expression of microRNA (miR)-155 induced by inflammasome activation (30).

Exploring the anti-tumor effects of resveratrol on glioblastoma and its role in modulating the inflammatory response within the tumor microenvironment demonstrated that resveratrol significantly reduced cell viability in glioblastoma cell lines LN-229 and U87-MG, inhibited cell proliferation and invasive migration, and promoted apoptosis. Network pharmacological analysis indicated that resveratrol’s anti-glioblastoma effects are closely linked to the Janus kinase (JAK)/signal transducer and activator of transcription (STAT) signaling pathway and inflammatory responses. Further validation through Western blot analysis revealed that resveratrol inhibits the over-activation of the NLRP3 inflammasome via the JAK2/STAT3 signaling pathway, with the JAK/STAT agonist RO8191 partially reversing this inhibition (75) (Table 1). 

The protective mechanisms of resveratrol were assessed against the pro-inflammatory effects of monomeric C-reactive protein (mCRP) in the field of neurodegeneration and Alzheimer’s disease. BV2 microglia were treated with mCRP, both with and without resveratrol, to assess its protective effects. The results showed that mCRP activated the nitric oxide (NO) pathway and the NLRP3 inflammasome, leading to increased cyclooxygenase-2 (COX-2) expression and the release of pro-inflammatory cytokines. Resveratrol effectively inhibited these inflammatory changes and enhanced the expression of anti-oxidant enzyme genes, including CAT and SOD2. Key defense mechanisms were identified, with resveratrol activating hub genes Sirt1 and Nfe2l2 while inhibiting the nuclear translocation of the NF-ĸB signaling pathway. Additionally, resveratrol provided protection against pro-inflammatory changes induced by mCRP in primary mixed glial cultures (76).

Another study explored the neuroprotective effects of exosomes derived from human neural stem cells (hNSCs) treated with resveratrol. The research demonstrated that treating SH-SY5Y cells, exposed to the neurotoxin MPP^+^, with resveratrol-hNSCs-exosome significantly improved cell viability, enhanced ATP production, and promoted mitochondrial biogenesis while reducing oxidative stress. The treatment also activated critical signaling pathways, including AMPK and Nrf2. Moreover, resveratrol-hNSCs-exosome efficiently suppressed neuroinflammation by inhibiting the NLRP3 inflammasome and decreasing the release of pro-inflammatory cytokines (77).

*Studies comprising both in vitro and in vivo parts*

*In vitro* findings of an investigation revealed that resveratrol suppressed the ATP-induced activation of NLRP3 and the cleavage of IL-1β in BV2 cells. Nicotinamide, a Sirt1 inhibitor, mitigated these effects. Moreover, the results of the *in vivo* part indicated that the administration of resveratrol to mice with sepsis-associated encephalopathy improved spatial memory, decreased apoptosis, and ionized calcium-binding adapter molecule 1 (Iba-1) positive microglia in the hippocampus, and inhibited NLRP3 expression and IL-1β cleavage (78). Treating BV2 cells exposed to lipopolysaccharide with polydatin, a glucoside of resveratrol, prevented inducible nitric oxide synthase (iNOS) expression and decreased NLRP3 inflammasome activation as well as microglial inflammation. The data from the *in vivo* part of the research illustrated that polydatin improved motor function and attenuated the production of NO, IL-1β, IL-6, and TNF-α (79).

Another study was designed to investigate the potential of resveratrol in mitigating secondary injury following spinal cord injury by targeting pyroptosis. The findings illustrated that death-associated protein kinase 1 (DAPK1) interacts with NLRP3, promoting pyroptosis through the NLRP3/caspase-1/Gasdermin D (GSDMD) pathway, and that knockdown of DAPK1 can inhibit this process. miR-124-3p was shown to negatively regulate DAPK1 levels, thereby reducing cell pyroptosis. Resveratrol was found to enhance the expression of miR-124-3p, leading to decreased DAPK1 levels and subsequent inhibition of pyroptosis via the NLRP3/caspase-1/GSDMD pathway. *In vivo* experiments demonstrated that resveratrol treatment reduced GSDMD-N levels in rats with spinal cord injury and promoted functional recovery (80).

*In vivo*

**Figure 1:**
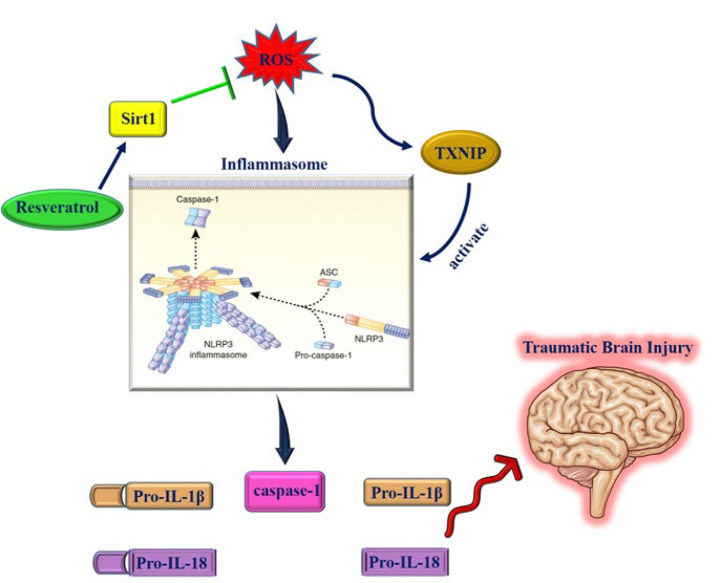
Therapeutic effects of resveratrol on traumatic brain injury (Images from https://smart.servier.com and https://commons.wikimedia.org/wiki/)

The administration of resveratrol to rats with brain injury after subarachnoid hemorrhage successfully inhibited NLRP3 expression, neutrophil infiltration, and microglia activation. It also reduced neurobehavioral impairment, cerebral inflammation, brain edema, and cortical apoptosis (81). Intraperitoneal injection of resveratrol to rats with cerebral ischemia/reperfusion injury increased autophagy while it reduced NLRP3 inflammasome-derived inflammation, cerebral infarct volume, and brain water content (82). The supplementation of resveratrol to rats with traumatic brain injury inhibited NLRP3 and caspase-1 activation, augmented Sirt1 activation, lowered the degree of traumatic brain injury, neuronspecific enolase, brain water content, inflammatory cytokines, and reactive oxygen species (ROS) (83) (Figure 1). 

Another preclinical study investigated the effects of resveratrol on mood alterations in rats subjected to social isolation stress. Results indicated that social isolation stress exposure led to significant anxiety and depression, with the social isolation stress plus normal saline group showing elevated levels of NLRP3, pro-caspase-1, ASC, and NF-κB. Notably, resveratrol treatment demonstrated antidepressant effects, outperforming fluoxetine in behavioral tests, and effectively reversed the NF-κB/NLRP3 signaling cascade in social isolation stress-exposed rats (84). 

In general, resveratrol has emerged as a promising modulator of neuroinflammation through its regulation of inflammasome activity, particularly the NLRP3 inflammasome, across various neurological disorders. The compound exerts protective effects by suppressing inflammasome activation, reducing oxidative stress, and inhibiting the release of pro-inflammatory cytokines such as IL-1β, TNF-α, and IL-6. Additionally, resveratrol enhances anti-oxidant defense mechanisms by up-regulating Nrf2 activity and increasing the expression of key anti-oxidant enzymes, including CAT and SOD. Beyond its direct anti-inflammatory effects, resveratrol also promotes mitochondrial function, glucose metabolism, and neuroprotection through pathways such as AMPK/Sirt1 signaling. Collectively, these mechanisms contribute to the attenuation of neuroinflammatory damage and cellular dysfunction across neurodegenerative and neuroinflammatory conditions.

One of the strengths of these findings is the consistent demonstration of the ability of resveratrol to modulate inflammasome-driven inflammation, highlighting its potential as a therapeutic agent for neuro-inflammatory disorders. However, while these studies confirm key aspects of the neuroprotective properties of resveratrol, some contradictions exist. For example, variations in experimental models, treatment dosages, and cell lines lead to differences in the magnitude of inflammasome inhibition and neuroprotection observed, suggesting the need for further standardization in experimental approaches. Moreover, while resveratrol has shown efficacy in conditions like Alzheimer’s disease, Parkinson’s disease, and traumatic brain injury, the lack of research on its role in stroke-related inflammasome modulation represents a significant gap. Considering the high prevalence of stroke and its association with inflammasome-mediated inflammation, future research should explore the potential advantages of resveratrol in alleviating post-stroke neuroinflammation and neuronal injury.

### Cardiovascular system

Inflammation is a major contributor to cardiovascular diseases (85, 86), and the inflammasome is a key mediator of this process (87). Chronic inflammation in cardiovascular disorders can result in the activation of the inflammasome in a variety of cell types, including macrophages and smooth muscle cells (88). Pro-inflammatory cytokines like IL-1β and IL-18 are released as a result of the activation of the inflammasome, which promotes inflammation and speeds up the development of cardiovascular illnesses (89). Additionally, the inflammasome activation results in the generation of active caspase-1, which promotes IL-1β and IL-18 maturation and secretion (90). These pro-inflammatory cytokines stimulate immune cell recruitment, atherosclerotic plaque formation, and endothelial dysfunction, provoking the progression of cardiovascular disorders (91). Additionally, persistent inflammasome activation could accelerate the development of heart failure, myocardial infarction, and other cardiovascular problems (92, 93). Therefore, understanding the role of inflammation and the inflammasome in cardiovascular diseases is crucial for the development of targeted therapies to mitigate their detrimental effects and improve patient outcomes.

*Studies comprising both in vitro and in vivo parts*

It has been demonstrated that resveratrol treatment improves cardiac hemodynamics and reduces mortality in mice with myocardial ischemia. The expression of senescence indicators (p16, p19, and p53), inflammasome markers (NLRP3 and caspase-1 p20), and NF-κB nuclear translocation were all successfully inhibited. Hence, resveratrol reduced the degree of infarction, fibrosis, and cell death. Moreover, the expression of IL-1β, IL-6, TNF-α, and IL-18 was also down-regulated by this substance. It also protected neonatal rat cardiomyocytes against hypoxia-induced senescence and apoptosis in the *in vitro* part of the investigation. Additionally, it had inhibitory effects on the expression of NLRP3 and caspase-1 p20 in neonatal rat cardiomyocytes, cardiac fibroblasts, and macrophages (31). Treating neonatal mouse cardiomyocytes exposed to isoproterenol with resveratrol resulted in lowered amounts of NLRP3, P20, and cleaved-IL-18. The findings of the *in vivo* part of the study revealed that intragastric administration of resveratrol to mice receiving isoproterenol attenuated inflammasome activation, cardiac macrophage infiltration, besides the expression of IL-6, TNF-α, monocyte chemoattractant protein (MCP)-1, and MCP-5 (94) (Table 2).

*In vivo*

It has been indicated that resveratrol pretreatment improved myocardial structure and reduced infarct volume and myocardial fibrosis, decreased trinitrotoluene and creatine kinase-myoglobin binding (CK-MB) levels, lowered the expression of PYD domains-containing protein (NALP)3 and caspase-1, as well as the activation of IL-1β and IL-18 (95). The supplementation of resveratrol to rats with hypercholesterolemia decreased vascular histopathological changes, declined serum levels of IL-1β, and attenuated the mRNA and protein expression levels of the three components of inflammasomes. Resveratrol revealed its anti-oxidant effect by increasing SOD and GPx activities and reducing malondialdehyde (MDA) amounts (96). The administration of resveratrol to animals with pulmonary embolism-associated cardiac injury increased miR223p and decreased the levels of metastasis-associated lung adenocarcinoma transcript 1 (MALAT1), NLRP3, ASC, caspase1, IL1β, and IL18 (97).

It has been reported that the administration of resveratrol to rats with chronic intermittent hypoxia decreased impaired cardiac structure and function, reduced oxidative stress and endoplasmic reticulum stress, as well as the induction of the NLRP3 inflammasome in cardiac tissue. Resveratrol appears to inhibit the NLRP3 inflammasome via activating AMPK, which can reduce the mammalian target of rapamycin (mTOR)/tristetraprolin (TTP)/NLRP3 mRNA signaling. Furthermore, resveratrol alleviated oxidative stress produced by chronic intermittent hypoxia by boosting anti-oxidant molecule expression via Nrf2. Resveratrol may also affect the Nrf2/HO-1 signaling pathway via AMPK (98). 

Furthermore, adding resveratrol to the diet of mice that received doxorubicin pointedly decreased the systolic blood pressure, systemic inflammation, as well as the expression of NLRP3, IL-18, IL-1β, and TNF-α (99). Likewise, the supplementation of resveratrol to rats with acute myocardial infarction ameliorated cardiac function, attenuated inflammatory cytokine levels, atrial interstitial fibrosis, NLRP3 inflammasome activity, transforming growth factor beta-1 (TGF-β1) production, and p-SMAD2/SMAD2 expression in cardiac tissue (100). 

Another study illustrated that resveratrol and regular exercise ameliorated hypertension-induced cardiac dysfunction by enhancing the expression of anti-oxidant genes, reducing systolic blood pressure, amending adrenergic and cholinergic altered responses of the right atrium and left papillary muscles, decreasing PTEN-induced putative kinase 1 (PINK1) expression, NLRP3 inflammasome activation, p-NF-κB expression, the mature-IL-1β/pro-IL-1β and cleaved-caspase-1/pro-caspase-1 ratio as well as the phosphorylation of p38 and Jun N-terminal kinase (JNK) (101). Similarly, the administration of resveratrol to rats with coronary micro-embolization-induced myocardial damage reduced myocardial injury, cardiac dysfunction, micro-infarct area, and cardiomyocyte pyroptosis by decreasing toll-like receptor 4 (TLR4)/myeloid differentiation primary response 88 (MyD88)/NF-κB signaling, NLRP3 inflammasome activation (102).

**Figure 2:**
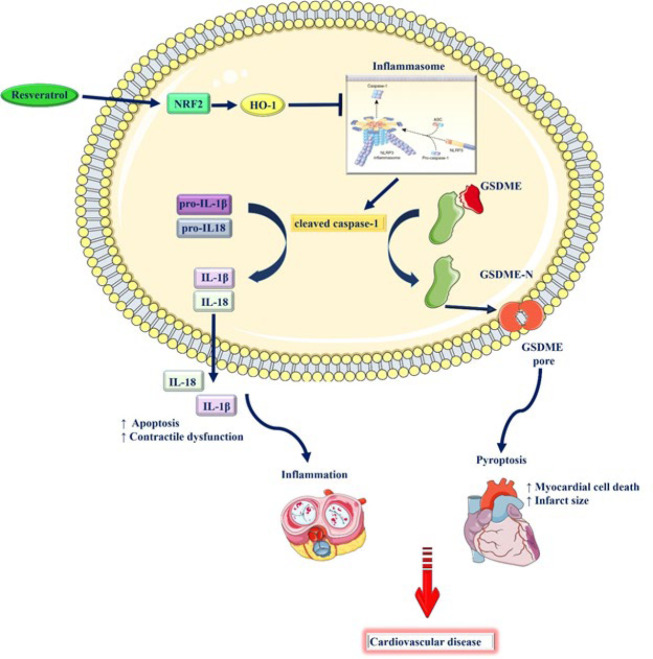
Proposed mechanism of cardioprotective effect of resveratrol as an inflammasome modulator (Images from https://smart.servier.com and https://commons.wikimedia.org/wiki/)

In summary, both *in vitro* and *in vivo* investigations showed that resveratrol has the ability to improve cardiac hemodynamics, reduce mortality, and protect against myocardial ischemia. Treatment with resveratrol has been demonstrated to suppress the expression of senescence indicators, inflammasome markers, and NF-κB nuclear translocation. These effects result in less infarction, fibrosis, and cell death. Furthermore, resveratrol inhibits the production of inflammatory cytokines such as IL-1β, IL-6, TNF-α, and IL-18, resulting in anti-inflammatory effects. Resveratrol pretreatment improved myocardial structure, decreased infarct volume, and reduced cardiac fibrosis in animal models (Figure 2). Additionally, it reduced cardiac macrophage infiltration, inflammasome activation, and pro-inflammatory factor expression. Besides, the administration of resveratrol consistently improves cardiac function, lowers inflammation, and lowers NLRP3 inflammasome activity in a number of cardiovascular disorders, including hypercholesterolemia, chronic intermittent hypoxia, doxorubicin-induced toxicity, and acute myocardial infarction. The preventive benefits of resveratrol against cardiac dysfunction and structural damage are attributed to its regulation of signaling pathways, including AMPK, Nrf2, and NF-κB. 

These studies exhibit certain strengths and limitations. The comprehensive approach combining *in vitro* and *in vivo* models strengthens the understanding of the effects of resveratrol. The consistency of its inflammasome-inhibiting properties across different cardiovascular diseases supports its therapeutic potential. Nonetheless, variability in dosages, administration methods, and disease models introduces limitations in translating findings to human applications. The bioavailability challenge of resveratrol remains a significant problem, potentially affecting its clinical efficacy. 

### Respiratory system

Inflammation plays a crucial role in respiratory system disorders (103, 104), and the inflammasome has appeared as a key player in regulating this inflammatory response (105). The respiratory system is continually exposed to environmental factors such as allergens, pollutants, and infections, all of which can cause an immunological response that leads to inflammation (106). Inflammasomes are triggered in response to danger signals emitted during tissue damage or infection, such as PAMPs and DAMPs. The activation of inflammasomes leads to the synthesis and release of pro-inflammatory cytokines such as IL-1β and IL-18 in respiratory system disorders (107). The cytokines contribute to the recruitment and activation of immune cells, such as neutrophils, leading to tissue damage and the exacerbation of respiratory symptoms (108). Asthma, chronic obstructive pulmonary disease, and lung fibrosis are just a few examples of respiratory disorders that can develop and worsen as a result of persistent inflammasome activation (109). Understanding the role of inflammation and the inflammasome in respiratory disorders is crucial for the development of targeted therapies aimed at modulating the immune response and alleviating the associated symptoms and tissue damage.

*In vitro*

The findings of *in vitro* research disclosed that resveratrol could reduce nickel-induced toxicity in BEAS-2B cells by reducing apoptosis and oxidative stress. It also declined the expression of IL-1β, IL-6, IL-8, and C-reactive protein, as well as the activity of p38 mitogen-activated protein kinase (MAPK), NF-κB, and NLRP3 (110) (Table 3).

*Studies comprising both in vitro and in vivo parts*

It has been shown that treating BEAS-2B cells exposed to *Mycoplasma pneumoniae *with polydatin reduced the rate of apoptosis. However, when NLRP3 was overexpressed, the protective effect of polydatin against *M. pneumoniae*-induced damage to BEAS-2B cells was reduced. Moreover, the results of the *in vivo* part of the study disclosed that the administration of polydatin to mice with *M. pneumoniae *infection reduced lung injury, attenuated the expression of IL-6, IL-1β, TNF-α, and MCP-1, lowered the levels of alpha-smooth muscle actin (α-SMA), TGF-β, collagen I, and collagen III, decreased the development of pulmonary fibrosis, activation of the NLRP3 inflammasome, and NF-κB pathway (111). It has been found that ﬁne particulate matter ((PM_2.5_)-induced cytotoxicity in BEAS-2B cells was ameliorated by treating with resveratrol through the reduction of NLRP3 inflammasome activity, IL-1β production, and autophagy. Resveratrol could also decrease lung fibrosis and inflammation, autophagy, and NLRP3 inflammasome activation in mice exposed to PM_2.5 _for five months (112).

Another study was designed to assess the protective role of resveratrol in mitigating lung injury caused by *Toxoplasma gondii* infection. Using both *in vivo* and *in vitro* models, researchers analyzed the effects of resveratrol on the TLR4/NF-κB/NLRP3 signaling pathway. The findings reveal that resveratrol inhibited NLRP3 inflammasome activation by suppressing TLR4 and NF-κB signaling, reducing IL-1β levels in bronchoalveolar lavage fluid (BALF), and preventing excessive inflammation. Resveratrol demonstrated a strong binding affinity to NLRP3, while the NLRP3 inhibitor CY-09 exhibited similar effects. Also, pretreatment with CY-09 diminished the protective effects of resveratrol (113). 

*In vivo*

The obtained data from an *in vivo* study showed that intraperitoneal injection of resveratrol to mice with lipopolysaccharide-induced acute lung injury decreased lung pathological damage, neutrophil infiltration, and lung edema by increasing Sirt1 mRNA and protein expression, decreasing IL-1β and IL-18 levels in BALF, reducing mRNA and protein expression of NLRP3, ASC, caspase-1, as well as NLRP3 inflammasome activation, ROS production, NF-kB p65 nuclear translocation, and NF-kB activity. Resveratrol also lowered the protein expression of TXNIP and the TXNIP-NLRP3 interaction (114). It has been shown that receiving resveratrol in mice with *Staphylococcus aureus *infection resulted in reduced mortality and lung injury. Resveratrol also attenuated cytokine levels in lung tissue and the BALF. Besides, mRNA and protein expression of NLRP3 and caspase1 were declined by resveratrol administration (115). The supplementation of resveratrol to rats with deep vein thrombosis ameliorated the lesions of lung tissue and inferior vena cava, reduced thrombosis, lowered the amounts of IL-1β and caspase-1, besides the expression of hypoxia-inducible factor 1-alpha (HIF-1α) and NLRP3 (116). 

Another study explored the protective effects of resveratrol against lipopolysaccharide-induced acute lung injury in mice. The findings indicated that resveratrol alleviated lung injury and inflammation by inhibiting ASC oligomerization and the activation of NLRP3 inflammasomes. Also, resveratrol reduced IL-1β and IL-18 levels in serum and BALF, suppressed caspase-1 expression, and limited macrophage pyroptosis. Moreover, it enhanced mitophagy by up-regulating autophagy-related 5 (Atg5), Beclin-1, Parkin, PINK1, and microtubule-associated proteins 1A/1B light chain 3B (LC3B-II), while PINK1 siRNA inhibited this process (117). 

To summarize, resveratrol has emerged as a promising therapeutic agent in respiratory disorders through its ability to modulate NLRP3 inflammasome activation. Across multiple studies, resveratrol consistently attenuates the production of IL-1β, IL-6, TNF-α, and other pro-inflammatory cytokines, suggesting that its anti-inflammatory effect is largely mediated by interfering with NF-κB and MAPK signaling. By suppressing inflammasome activation, resveratrol mitigates oxidative stress, apoptosis, and pulmonary fibrosis, demonstrating protective effects against environmental pollutants, infections, and acute lung injury.

One key mechanism underlying the effects of resveratrol is its role in autophagy and mitophagy, particularly through Sirt1, PINK1/Parkin, and Beclin-1 pathways. By enhancing cellular detoxification and mitochondrial quality control, resveratrol provides sustained protection against chronic inflammation-induced lung damage. Besides, its ability to bind directly to NLRP3 may indicate a novel mechanism of inflammasome regulation, which could be further explored as a direct pharmacological approach for inflammasome-associated disorders.

Despite these promising findings, variability among studies suggests that the extent of influence of resveratrol on the inflammasome may depend on disease context and dosage. This discrepancy might stem from differences in disease models, administration routes, or timing of intervention. Particularly, the pharmacokinetics and bioavailability of resveratrol remain major limitations for clinical translation, requiring optimized formulations for effective respiratory therapy.

### Renal system

Inflammatory processes play a pivotal role in the development and progression of renal system disorders (118, 119), and the inflammasome is one of the essential factors (120). In renal disorders such as chronic kidney disease, inflammasome activation and subsequent cytokine release contribute to chronic inflammation and tissue damage (121, 122). This process can further promote fibrosis, impair renal function, and even contribute to the progression of renal disorders (123). Understanding the complex interaction between inflammation, the inflammasome, and renal system disorders is crucial for the development of targeted therapies to modulate the inflammatory response and maintain renal function.

*In vitro*

Treating HK-2 cells with resveratrol-loaded nanoparticles induced autophagy and decreased NLRP3 inflammasome and IL-1β secretion (124) (Table 3).

*Studies comprising both in vitro and in vivo parts*

Resveratrol was investigated for its potential effects on renal cell carcinoma and its underlying molecular mechanisms. The findings indicate that resveratrol effectively restricted renal cell carcinoma tumor growth by reducing proliferation, migration, and invasion while enhancing apoptotic activity in tumor cells, both *in vivo* and *in vitro*. A key aspect of its mechanism involves the suppression of NLRP3 expression and its associated downstream genes (125).

*In vivo*

In the contrast-induced nephropathy model in rats, the administration of resveratrol could remarkably attenuate tubule injury, decrease the levels of blood urea nitrogen (BUN), creatinine, neutrophil gelatinase-associated lipocalin (NGAL), IL-1β, caspase-3, and NLPR3 (126).

In summary, resveratrol has emerged as a promising therapeutic agent for controlling inflammasomes in renal disorders, primarily by suppressing NLRP3 activation and its downstream inflammatory mediators. Across *in vitro* and *in vivo* models, resveratrol demonstrates protective effects by inducing autophagy, mitigating inflammatory cytokine secretion, and reducing renal cell carcinoma progression. These mechanisms collectively suggest that resveratrol exerts anti-inflammatory and anti-tumor effects through modulating inflammasome activity and apoptotic pathways.

A more particular way to describe the effect of resveratrol-loaded nanoparticles is that they enhance or amplify their therapeutic potential. The nanoparticle formulation improves bioavailability and optimizes inflammasome suppression, making resveratrol more effective in targeting renal inflammation. Moreover, the ability of resveratrol to target multiple pathways, such as autophagy induction and cytokine suppression, indicates its broader potential as a renal-protective agent beyond inflammasome control.

However, these studies are limited in number, with only a few investigations evaluating their effects in both cellular and animal models. While the available data suggest promising anti-inflammatory and anti-tumor benefits, further research is essential to determine optimal dosage, long-term efficacy, and potential translation into clinical applications. Also, while resveratrol suppression of inflammasomes appears beneficial, the variability in experimental models and the lack of large-scale studies prevent definitive conclusions. Future studies should explore its combinatorial potential with existing renal therapies and assess whether its nanoparticle formulations could maximize therapeutic effectiveness. 

### Digestive system

Inflammation plays a crucial role in the digestive system, which encompasses the liver, gut, and other organs (127, 128). Inflammation in the liver can be caused by a variety of reasons, such as infection, alcohol addiction, or metabolic problems (55, 129). Inflammasome activation in liver cells, such as hepatocytes and Kupffer cells, can result in the release of pro-inflammatory cytokines such as IL-1β and IL-18, which contribute to liver inflammation and damage (130).

Inflammasomes have been linked to the pathophysiology of inflammatory bowel diseases (131). Activation of inflammasomes in the small intestine can result in the production of IL-1β and IL-18 (132). Increased intestinal permeability, immune cell recruitment, and tissue damage occur as a result, increasing the inflammatory response (133). Inflammasomes also influence the composition and diversity of the gut microbiota, which can contribute to intestinal inflammation (134). Generally, the role of inflammation and inflammasomes in the digestive system is complex. Understanding the mechanisms underlying inflammasome activation and regulation can provide insights into the development and progression of various digestive disorders. Targeting inflammasomes and their associated pathways may hold therapeutic potential for managing inflammation-related conditions in the liver, intestine, and other digestive organs.

*In vitro*

To assess the effect of resveratrol on ulcerative colitis progression, researchers established an *in vitro* inflammation model using HT29 colon cancer cells exposed to ATP and lipopolysaccharide. Findings revealed that lipopolysaccharide/ATP exposure up-regulated pyroptosis-related proteins and inflammatory cytokines while enhancing NF-κB pathway activation. Resveratrol supplementation counteracted these effects, reducing pyroptosis and inflammatory responses by suppressing ASC, caspase-1, IL-6, IL-1β, IL-18, NLRP3, and TNF-α expression. Its mechanism was linked to NF-κB pathway inhibition (135).

Exploring the molecular mechanisms of PM_2.5_-induced liver fibrosis and the potential protective role of resveratrol in ameliorating hepatic injury demonstrated that PM_2.5 _exposure caused cytotoxicity and oxidative stress in mHSCs, elevating ROS and MDA levels while impairing anti-oxidant defenses. It also increased expression of fibrotic markers (α-SMA, collagen I, and collagen III) as well as cleaved-caspase1, IL-1β, NF-κB, and NLRP3. However, resveratrol supplementation reversed these effects by enhancing SOD activity, reducing fibrotic protein expression, and regulating Sirt1, thereby suppressing inflammatory and oxidative stress pathways (136).

*Studies comprising both in vitro and in vivo parts*

One strategy to tackle pharmacokinetic limitations and boost the biological activities of resveratrol is designing synthetic structural analogs (137, 138). In this context, the effect of the resveratrol analog, 2-methoxyl-3,6-dihydroxyl-IRA, was examined on the colitis model. The results indicated that resveratrol increased the expression of genes downstream of Nrf2 in both *in vitro* (LS174T cells) and *in vivo* parts. In addition, resveratrol enhanced the activation of the Nrf2 pathway and decreased colitis inflammation score and inflammatory markers (IL-6 and TNF-α), in addition to NLRP3 inflammasome (139). Furthermore, treating Caco-2 cells exposed to lipopolysaccharide increased Sirt1 activity and, meanwhile, decreased the levels of NLRP3, caspase-1, and ASC. Additionally, the intraperitoneal injection of resveratrol into rats with sepsis reduced the ASC-positive area in the intestinal epithelium, besides the expressions of NLRP3 and caspase-1 (140).

With severe acute pancreatitis-induced models in rats and pancreatic cells, researchers found that resveratrol treatment improved pancreatic microcirculation, reduced oxidative stress, and suppressed inflammasome-mediated pyroptosis. Resveratrol inhibited NLRP3 inflammasome activation, decreased pro-inflammatory markers, and enhanced mitochondrial membrane stability, thereby promoting cellular resilience. In addition, resveratrol facilitated mitochondrial biogenesis through Sirt1/PGC-1α signaling (141).

*In vivo*

It has been claimed that resveratrol amended hepatic metaflammation in mice by improving glucose control, decreasing serum and liver triglyceride amounts, and lowering hepatic levels of IL-1, IL-6, and TNF-α, besides reducing the expression of ASC and NLRP3 (19). The administration of resveratrol to old mice reduced hepatic amounts of IL-1β, COX-2, and TNF-α, as well as the mRNA levels of NALP-3 inflammasome, ASC, caspase-1, NALP-1, and NALP-3 (142). Pretreatment with resveratrol could amend inflammation stimulated by intestinal ischemia-reperfusion in rats by reducing intestinal injury, decreasing the levels of IL-1β, IL-18, and TNF-α, besides mast cells and NLRP3 inflammasome activation (143) (Table 4). 

It has been illustrated that the administration of resveratrol to diabetic rats could significantly reduce blood glucose, serum insulin, and triglyceride levels. Resveratrol also decreased the amounts of caspase-1 and NLRP3, but this reduction was not significant (144). Likewise, the supplementation of resveratrol to mice with inflammatory bowel disease reduced bowel inflammation, besides the expression of NLRP3 and IL-1β (145). In the acute heat stress model, resveratrol amended inflammation in the jejunum of ducks by increasing the villus height/crypt depth ratio, improving the number of goblet cells, activating Sirt1-NRF1/NRF2 signaling pathways, and augmenting the levels of ATP, SOD, and CAT. Resveratrol was also found to decrease jejunum histopathological damage as well as the levels of NLRP3, caspase1 p20, and IL-1β (146). 

A study was carried out to determine the protective mechanisms of resveratrol against aflatoxin B1-induced ileum injury in ducks, and it was found that aflatoxin B1 significantly disrupted the intestinal anti-oxidant system, leading to deoxyribonucleic acid (DNA) damage, mitochondrial dysfunction, inflammation, and oxidative damage. Aflatoxin B1 also up-regulated key detoxification genes while suppressing protective pathways such as Nrf2, causing NF-κB activation. However, resveratrol supplementation reversed these harmful effects, reducing aflatoxin B1-DNA adducts and restoring oxidative balance by regulating the NF-κB/NLRP3 and Nrf2/ Kelch-like ECH-associated protein 1 (Keap1) pathways (147). 

The administration of resveratrol to rats with increased circulating asymmetric dimethylarginine pointedly decreased the NLRP3 inflammasome in the ileum and plasma and the levels of IL-1α and IL-6 in the ileum (148). Receiving resveratrol could reduce the protein levels of NLRP3, ASC, caspase, and IL-1β in the jejunum of rats with a high-fat diet. The primary target of resveratrol was the gut microbiota, and resveratrol enhanced lipid homeostasis via attenuating Farnesoid X receptor-stimulated gut scavenger receptor class B type 1 (SR-B1) increase (149).

Finally, resveratrol has emerged as a promising therapeutic compound for controlling inflammasome activation and alleviating digestive system disorders. Its ability to modulate key inflammatory pathways, particularly the NLRP3 inflammasome and NF-κB signaling, highlights its complex protective mechanisms across various conditions affecting intestinal and hepatic health. Resveratrol functions primarily by suppressing pyroptosis-related markers, reducing oxidative stress, and restoring cellular homeostasis. It enhances anti-oxidant defenses through the Nrf2 pathway and improves mitochondrial stability, ultimately preventing inflammasome-induced tissue damage. Furthermore, the effect of resveratrol on gut microbiota suggests a broader regulatory role in maintaining intestinal balance, which may contribute to metabolic health beyond direct anti-inflammatory effects.

A significant implication of these findings is the ability of resveratrol to counteract inflammation-driven disorders at multiple levels—cellular, molecular, and systemic. By stabilizing intestinal epithelial function, resveratrol may not only lessen ulcerative colitis progression but also play a preventive role in metabolic and age-related intestinal diseases. Its interactions with pathways such as Sirt1-p66Shc-NLRP3 and NF-κB/Nrf2 further underscore its therapeutic potential in conditions ranging from sepsis-induced intestinal inflammation to toxin-mediated gut injury. However, despite these promising results, several limitations should be considered. The translation of *in vitro* and *in vivo* findings into clinical applications remains challenging, requiring further validation through well-controlled human studies (150). In addition, dosage and bioavailability optimization and long-term efficacy need to be explored to ensure maximum therapeutic benefits while minimizing potential side effects.

Future research should investigate the synergy of resveratrol with other natural bioactive compounds or standard-of-care pharmaceutical products, aiming for a more comprehensive approach to inflammasome regulation. Exploring personalized dietary strategies incorporating resveratrol could also open new opportunities for managing gut-related inflammatory disorders, potentially offering targeted treatments based on individual inflammatory profiles.

### Reproductive system

The inflammasome plays a crucial role in reproductive health by regulating inflammation and cell death mechanisms. Dysregulation of inflammasome activation, particularly involving NLRP3, has been implicated in various reproductive disorders, including infertility, endometriosis, preeclampsia, and recurrent pregnancy loss. Excessive inflammasome activity disrupts the balance between pro- and anti-inflammatory signals, leading to poor oocyte quality, impaired sperm function, and placental inflammation (151). 

*In vitro*

Exploring the effect of oxidative stress on placental trophoblasts, focusing on NLRP1 activation and autophagy while assessing the protective effects of resveratrol, disclosed that the oxidative damage model using H_2_O_2_ exposure led to increased cellular apoptosis, oxidative stress markers, and inflammasome activation. Elevated levels of Beclin-1, caspase-1, IL-1β, LC3, NLRP1, and confirmed that oxidative stress triggers inflammatory and autophagic responses. Resveratrol treatment effectively counteracted these changes by enhancing cell survival, reducing oxidative stress, and suppressing inflammasome activity (152).

*Studies comprising both in vitro and in vivo parts*

The protective role of resveratrol was tested against male reproductive toxicity induced by 3-monochloropropane-1,2-diol infertility by using a rat model. 3-Monochloropropane-1,2-diol exposure led to significant reductions in epididymal and testicular weights, disrupted spermatogenesis, and caused histological abnormalities. Resveratrol co-treatment mitigated these effects by preserving Leydig and Sertoli cell populations. Resveratrol also suppressed inflammasome activation (NLRP3), reduced endoplasmic reticulum stress (poly (ADP-ribose) polymerases (PARP)), and restored autophagy-mediated lysosomal function (p62 and LC3BII). Furthermore, 3-monochloropropane-1,2-diol exposure increased steroidogenesis-related protein levels (steroidogenic acute regulatory and CYP11A1), but resveratrol normalized steroidogenic acute regulatory expression. Resveratrol also counteracted pro-inflammatory responses by decreasing cytokine levels (IL-1β, IL-18) and modulating CD206 expression (153). 

*In vivo*

In a rat model of polycystic ovary syndrome induced by high-fat diet and letrozole, resveratrol intervention improved metabolic and reproductive disturbances, including obesity, estrous cycle irregularities, and ovulation disorders. Resveratrol reduced serum testosterone and lipopolysaccharide levels while down-regulating key inflammatory markers such as IL-6, TNF-α, and NLRP3. Moreover, pyroptosis-related proteins, including cleaved-caspase-1, GSDMD, IL-1β, and IL-18, were significantly suppressed (154) (Table 4). 

The effects of monosodium glutamate and resveratrol were examined on male reproductive health. Exposure to monosodium glutamate resulted in lower luteinizing hormone (LH), follicle-stimulating hormone (FSH), and testosterone levels, alongside diminished sperm quality. Histological analysis displayed notable testicular degeneration, and biochemical assessments showed reduced anti-oxidant defenses (CAT and SOD) and increased lipid peroxidation (MDA). Furthermore, monosodium glutamate exposure triggered inflammatory and cell death pathways by up-regulating caspase-3 and NLRP3. Resveratrol supplementation effectively reversed these harmful effects by stabilizing hormonal levels, enhancing sperm health, lowering oxidative stress, and suppressing apoptotic and pyroptotic activity. Besides, resveratrol promoted the expression of Ki-67 (155). 

Briefly, resveratrol has demonstrated remarkable potential in regulating inflammasome activation and ameliorating reproductive system disorders through its anti-inflammatory and anti-oxidant properties. Its ability to modulate key molecular pathways, particularly the NLRP3 inflammasome and pyroptosis axis, provides a strong basis for its therapeutic applications in conditions such as polycystic ovary syndrome, male infertility, and placental oxidative damage. By inhibiting inflammasome activity and reducing oxidative stress, resveratrol restores cellular homeostasis, improves reproductive function, and enhances mitochondrial integrity. In ovarian granulosa cells, resveratrol counteracts inflammation-driven disruptions in follicular development by suppressing NLRP3/GSDMD/caspase-1-mediated pyroptosis, proposing possibilities for managing polycystic ovary syndrome-related infertility. Furthermore, its protective effects extend to male reproductive health, where it preserves testicular function, balances steroidogenesis, and attenuates cellular damage induced by environmental toxins such as monosodium glutamate and 3-Monochloropropane-1,2-diol.

A significant implication of these findings is the advantageous role of resveratrol in addressing broader reproductive health concerns linked to inflammation and oxidative stress. Beyond isolated conditions, resveratrol may contribute to overall fertility preservation by optimizing endocrine balance, improving microcirculatory function, and enhancing mitochondrial biogenesis. However, while existing studies provide strong information, certain limitations remain. The majority of findings are derived from experimental models, highlighting the need for well-controlled clinical trials to validate the efficacy of resveratrol in human reproductive health. Moreover, the variability in effective dosages and bioavailability challenges its widespread application, necessitating further research on optimal therapeutic strategies.

Future studies should investigate the synergistic interactions of resveratrol with other bioactive compounds to maximize its anti-inflammatory potential. Exploring its role in reproductive aging and hormone regulation may offer new perspectives on fertility preservation and endocrine health. Also, refining targeted drug delivery systems for resveratrol could improve its bioavailability and therapeutic effectiveness, making it a practical candidate for integrative reproductive medicine.

### Limitations of resveratrol as a therapeutic agent

While resveratrol has demonstrated significant potential in modulating inflammasomes and inflammation, several limitations must be acknowledged before drawing definitive conclusions about its therapeutic application: Resveratrol has low bioavailability due to rapid metabolism and poor absorption, limiting its effectiveness *in vivo*. Efforts to enhance delivery through nanoparticles, liposomes, and structural modifications are ongoing but require further validation. The results of resveratrol studies vary significantly based on experimental conditions, including cell types, animal models, and dosages. These differences can make it challenging to translate findings into clinical applications. Despite promising preclinical data, human studies on the effects of resveratrol on inflammasome modulation remain limited. Most clinical trials have focused on resveratrol’s general anti-inflammatory properties, and more targeted investigations are needed to confirm its efficacy in inflammasome-related diseases.

### Limitations of the present review

While this review aimed to provide a comprehensive analysis of the role of resveratrol in inflammasome modulation, certain limitations must be acknowledged: The search was restricted to peer-reviewed studies available in English, which may exclude relevant research published in languages other than English. Furthermore, selection was based on predefined keywords, and there is always a possibility that some pertinent studies were inadvertently omitted. Studies with positive findings are more likely to be published, potentially leading to an overrepresentation of research supporting the advantages of resveratrol while underestimating conflicting or negative results. The possibility of such a publication bias cannot be precluded in reviews. Finally, due to the broad scope of the review, the discussion may not provide exhaustive detail on individual mechanisms or experimental variations. Some aspects, such as hormones and tumors, would require separate in-depth reviews to ensure proper coverage.

## Conclusion

Resveratrol has emerged as a promising naturally occurring inflammasome modulator with significant implications for health and disease. Resveratrol exerts its effects through the suppression of inflammasome activation across multiple organ systems, including the nervous, cardiovascular, respiratory, digestive, renal, and reproductive systems. This suppressive effect can lead to the reduction of pro-inflammatory cytokine production and modulation of key molecular pathways such as NF-κB, Nrf2, and Sirt1. Resveratrol’s ability to regulate oxidative stress, mitochondrial function, and cellular homeostasis highlights its broad therapeutic potential.

The collective evidence from *in vitro* and *in vivo* studies supports the effectiveness of resveratrol in alleviating inflammation-driven tissue damage, thereby improving cellular resilience and protecting against various diseases. Resveratrol’s anti-inflammatory and anti-oxidant properties contribute to neuroprotection, cardiovascular health, pulmonary defense, gastrointestinal stability, renal function, and reproductive health. Moreover, the ability of this phytochemical to interact with inflammasome components and modulate immune responses underscores its potential as a therapeutic agent for inflammasome-related disorders.

Despite the aforementioned beneficial effects, significant challenges remain in converting these findings into clinical applications. The low bioavailability, variability in study models, and lack of standardized dosing of resveratrol present challenges that must be overcome through optimized drug delivery systems, targeted formulations, and further well-controlled human studies. Besides, the inconsistencies observed in experimental outcomes emphasize the need for standardized protocols and deeper investigations.

Despite these limitations, the consistent ability of resveratrol to regulate inflammasome-mediated inflammation reinforces its potential value in disease prevention and treatment. Future research should focus on refining its pharmacokinetics, exploring combination therapies, and expanding investigations into less-studied disease contexts. With continued advancements, resveratrol holds strong potential to drive innovative strategies in inflammatory disease management and overall health improvement.

**Table 1:** Resveratrol's role as an inflammasome modulator on nervous system disorders

Study design		Doses/Duration	Results	Ref.
*In vitro*				
BV2 cells		10 and 50 nM, 24 hr	↓ Aβ-induced proliferation and activation of BV-2 cells, IL-6, TNF-α, expression of cleaved caspase-1 and IL-1β, phosphorylation of NF-κB, degradation of IkBα, TXNIP/TRX/NLRP3 pathway	(73)
SH-SY5Y cells		10 μM, 48 hr	↑Cell viability, mRNA levels of Nrf2, CAT, and HO-1, activity of Nrf2, SOD, and GPx, GSH ↓TNF-α, IL-1β, IL-18, mRNA levels of IKKα, IKKβ, p65, NLRP3, and caspase-1, activity of caspase-1	(20)
HMC3 human microglia cells		100 μM, 24 hr	↑ Insulin-like growth factor 1 release, glucose uptake ↓Inflammasome activity, mitochondrial activity, cellular metabolism	(74)
N9 microglial cells		-	↑AMPK/Sirt1 pathways ↓Oxidative stress, pro-inflammatory cytokine production, pyroptotic cell death, miR-155 expression, NLRP3 activity, NF-κB signaling	(30)
LN-229 and U87-MG cells		20 μM, 48 hr	↑ Apoptosis ↓Cell viability, cell proliferation, invasive migration, NLRP3 inflammasome	(75)
BV2 cells		1-50 µM, 24 hr	↑ Expression of CAT and SOD2, activating Sirt1 and Nfe2l2 ↓ NO, NLRP3 inflammasome, COX-2, NF-κB	(76)
SH-SY5Y cells		10 µM, 48 hr	↑ Cell viability, ATP production, mitochondrial biogenesis, AMPK, Nrf2 ↓ Oxidative stress, neuro-inflammation, NLRP3 inflammasome, pro-inflammatory cytokines	(77)
*In vitro *plus* in vivo*				
*In vitro*, BV2 cells		15 and 30 µM	↓NLRP3 expression and IL-1β cleavage	(78)
*In vivo*, Male C57BL/6 mice		10 and 30 mg/kg, abdominal infusions	↑Spatial memory ↓Apoptosis, iba-1 positive microglia in the hippocampus, NLRP3 expression and IL-1β cleavage	
*In vitro*, BV2 cells		1, 2, and 4 μM, 24 hr	↓ NLRP3 inflammasome activation, microglial inflammation, iNOS expression	(79)
*In vivo*, Male Sprague-Dawley rats		20, 40 mg/kg, IP	↑Motor function ↓Production of NO, IL-1β, IL-6, and TNF-α	
*In vitro*, BV2 cells		40 μM, 1 hr	↑ Expression of miR-124-3p, ↑ DAPK1 levels, pyroptosis	(80)
*In vivo*, Female Sprague–Dawley rats		200 mg/kg, 14 days, IP	↑ Functional recovery ↓GSDMD-N	
*In vivo*				
Male Sprague-Dawley rats	30, 60, and 90 mg/kg, IP		↓Neurobehavioral impairment, cerebral inflammation, brain edema, cortical apoptosis, NLRP3 expression, microglia activation, and neutrophil infiltration	(81)
Male Sprague-Dawley rats	100 mg/kg, IP		↑ Autophagy ↓NLRP3 inflammasome-derived inflammation, cerebral infarct volume, brain water content	(82)
Male Sprague-Dawley rats	100 mg/kg, IP		↑Sirt1 activation ↓Degree of traumatic brain injury, neuron‑specific enolase, brain water content, inflammatory cytokines, ROS, NLRP3, and caspase-1 activation	(83)
Rats	20, 40, and 80 mg/kg, 4 weeks, IP		↑ Antidepressant effects ↓ NF-κB/NLRP3 signaling cascade	(84)

ATP: adenosine triphosphate; CAT: catalase; COX-2: cyclooxygenase-2; DAPK1: death-associated protein kinase 1; GPx: glutathione peroxidase; GSDMD: Gasdermin; GSH: glutathione; HO-1: heme oxygenase-1; IkBα: inhibitor of kappa B alpha; IKKα: inhibitor of nuclear factor Kappa-B kinase subunit alpha; IL: interleukin; mRNA: messenger ribonucleic acid; NF-κB: nuclear factor-kappa B; NLRP3: nucleotide-binding oligomerization domain-like receptor family pyrin domain-containing 3; NO: nitric oxide; Nrf2: nuclear factor erythroid 2-related factor 2; Sirt1: sirtuin 1; SOD: superoxide dismutase; TNF-α: tumor necrosis factor-alpha; TRX: Thioredoxin; TXNIP: Thioredoxin interacting protein

**Table 2:** Resveratrol's role as an inflammasome modulator on cardiovascular system disorders

Study design	Doses/Duration	Results	Ref.
*In vitro *plus* In vivo*			
*In vitro*, neonatal rat cardiomyocytes, cardiac fibroblasts, and macrophages	-	↓Hypoxia-induced neonatal rat cardiomyocytes senescence and apoptosis, expression of NLRP3 and caspase-1 p20	(31)
*In vivo*, Male C57BL/6J mice	320 mg/kg, 3 weeks, gavage	↑ Cardiac hemodynamics ↓Mortality, expression of p53, p16, p19, NLRP3, caspase-1 p20, IL-1β, IL-18, and TNF-α, nuclear translocation of NF-κB, infarction area, fibrosis, and cell apoptosis	
*In vitro*, neonatal mouse cardiomyocytes	100 nmol/l, 10 μmol/1, 30 min	↓ NLRP3, P20	(94)
*In vivo*, Male C57BL/6J mice	20 mg/kg, 4 days, intragastric	↓Inflammasome activation, cardiac macrophage infiltration, expression of IL-6, TNF-α, MCP-1, and MCP-5	
*In vivo*			
Male Sprague-Dawley rats	2.5, 5, 10 mg/kg, IP	↑Myocardial structure ↓Infarct volume and myocardial fibrosis, trinitrotoluene and CK-MB levels, expression of NALP3 and caspase-1, activation of IL-1β and IL-18	(95)
Male Sprague-Dawley rats	50 mg/kg, 5 weeks, PO	↑ SOD and GPx activities ↓Vascular histopathological changes, serum levels of IL-1β, mRNA, and protein expression, levels of the three components of inflammasomes, MDA amounts	(96)
Male Sprague-Dawley rats	30 mg/kg, 5 weeks, gavage	↑ AMPK activation, Nrf2 ↓Impaired cardiac structure and function, oxidative stress, endoplasmic reticulum stress, NLRP3 inflammasome induction, mTOR/TTP/NLRP3 mRNA signaling	(98)
Male C57Bl/6N mice	320 mg/kg, 5 weeks, PO	↓Systolic blood pressure, systemic inflammation, expression of NLRP3, IL-18, IL-1β, TNF-α	(99)
Male Sprague-Dawley rats	50 mg/kg, 45 days, gavage	↑Cardiac function ↓Inflammatory cytokine levels, atrial interstitial fibrosis, NLRP3 inflammasome activity, TGF-β1 production, and p-SMAD2/SMAD2 expression	(100)
Male Wistar rats	6 weeks	↑ Expression of antioxidant genes ↓ Systolic blood pressure, adrenergic and cholinergic altered responses of the right atrium and left papillary muscles, PINK1 expression, NLRP3 inflammasome activation, p-NF-κB expression, mature-IL-1β/pro-IL-1β and cleaved-caspase-1/pro-caspase-1 ratio, phosphorylation of p38 and JNK	(101)
Male Sprague-Dawley rats	25 and 50 mg/kg	↓Myocardial injury, cardiac dysfunction, microinfarct area, cardiomyocyte pyroptosis, TLR4/MyD88/NF-κB signaling, NLRP3 inflammasome activation	(102)

Akt: protein kinase B; AMPK: adenosine monophosphate-activated protein kinase; ASC: apoptosis speck-like protein; CK-MB: creatine kinase-myoglobin binding; GPx: glutathione peroxidase; IL: interleukin; JNK: Jun N-terminal kinase; MALAT1: metastasis-associated lung adenocarcinoma transcript 1; MCP: monocyte chemoattractant protein; MDA: malondialdehyde; miR223p: microRNA-22-3p; mRNA: messenger ribonucleic acid; mTOR: rapamycin; MyD88: myeloid differentiation primary response 88; NF-κB: nuclear factor-kappa B; NLRP: nucleotide-binding oligomerization domain-like receptor family pyrin domain-containing; Nrf2: nuclear factor erythroid 2-related factor 2; SOD: superoxide dismutase; TGF-β1: transforming growth factor beta-1; TLR4: toll-like receptor 4; TNF-α: tumor necrosis factor-alpha; TTP: tristetraprolin

**Table 3:** Resveratrol's role as an inflammasome modulator on respiratory and renal systems disorders

Study design	Doses/Duration	Results	Ref.
Respiratory system			
*In vitro*			
BEAS‐2B cells	10, 20, 40 μM, 24 hr	↓ Apoptosis, oxidative stress, expression of IL-1β, IL-6, IL-8, C-reactive protein, activity of MAPK, NF‐κB, NLRP3	(110)
*In vitro *plus* In vivo*			
*In vitro*, BEAS‐2B cells	100 μM, 24 hr	↓Apoptosis	(111)
*In vivo*, BALB/c mice	100 mg/kg, 3 weeks, IG	↓Lung injury, expression of IL‐6, IL‐1β, TNF‐α, MCP‐1, α‐SMA, TGF-β, collagen I, collagen III, development of pulmonary fibrosis, activation of the NLRP3 inflammasome, NFκB	
*In vitro*, BEAS‐2B cells	0–10 μmol/L, 24 hr	↓Cytotoxicity, NLRP3 inflammasome activity, IL-1β production, autophagy	(112)
*In vivo*, Male C57BL/6J mice	50 and 100 mg/kg, every 2 days, gavage	↓ Lung fibrosis, inflammation, autophagy, NLRP3 inflammasome activation	
*In vivo*			
Male C57BL6 mice	30 mg/kg, IP	↑ Sirt1 mRNA and protein expression ↓ Lung pathological damage, lung Edema, neutrophil infiltration, IL-1β and IL-18 in BALF, mRNA and protein expression of NLRP3, ASC, caspase-1, NLRP3 inflammasome activation, ROS, NF-kB p65 nuclear translocation, NF-kB activity, protein expression of TXNIP, TXNIP-NLRP3 interaction	(114)
Female C57BL/6J mice	30 mg/kg, subcutaneous	↓ Mortality, lung injury, cytokine levels in lung tissue, and BALF, mRNA, and protein expression of NLRP3 and caspase‑1	(115)
Sprague-Dawley rats	60 mg/kg	↓Lesions of lung tissue and inferior vena cava, thrombosis, IL-1β, caspase-1, expression of HIF-1α and NLRP3	(116)
C57BL/6 J mice	30 µM, 1 hr before the liposaccharide treatment, IP	↑ Mitophagy, Atg5, Beclin-1, Parkin, PINK1, LC3B-II ↓ Lung injury, inflammation, ASC oligomerization, activation of NLRP3 inflammasomes, IL-1β and IL-18 in serum and BALF, caspase-1 expression, macrophage pyroptosis	(117)
Renal system			
*In vitro*			
HK-2 cells		↑ Autophagy ↓ NLRP3 inflammasome and IL-1β secretion	(124)
*In vitro *plus* in vivo*			
*In vitro*, Human RCC lines ACHN and 786-O	25, 50, 100 and μM, 12–24 hr	↑Bax/Bcl-2 ratio ↓ Cell viability, migration, invasion, expression of NLRP3	(125)
*In vivo*, Male nude mice	60 mg/kg, 40 days, gavage	↓Tumor volume and weight, expression of NLRP3	
*In vivo*			
Male Sprague-Dawley rats	30 mg/kg, IV	↓ BUN, creatinine, NGAL, IL-1β, caspase-3, and NLPR3, tubule injury	(126)

α-SMA: alpha-smooth muscle actin; ASC: apoptosis-associated speck-like protein containing a CARD domain; BALF: broncho-alveolar lavage fluid; Bcl-2: B-cell lymphoma protein 2; Bax: Bcl-2)-associated X; HIF-1α: hypoxia-inducible factor 1-alpha; IL: interleukin; LC3B-II: microtubule-associated proteins 1A/1B light chain 3B; MAPK: p38 mitogen-activated protein kinase; MCP-1: monocyte chemoattractant protein-1; mRNA: messenger ribonucleic acid; NF-κB: nuclear factor-kappa B; NLRP: nucleotide-binding oligomerization domain-like receptor family pyrin domain-containing; PINK1: PTEN induced putative kinase 1; Sirt1: Sirtuin 1; TGF-β: transforming growth factor-beta; TNF-α: tumor necrosis factor-alpha; TXNIP: Thioredoxin interacting protein

**Table 4:** Resveratrol's role as an inflammasome modulator in digestive system and reproductive system disorders

Study design		Doses/Duration	Results	Ref.
Digestive system				
*In vitro *				
HT29 colon cancer cells		25–50 μmol/l, 28 hr	↓ Pyroptosis, inflammatory responses, expression of ASC, caspase-1, IL-1β, IL-6, IL-18, NLRP3, TNF-α, NF-κB pathway	(135)
mHSCs cells		0, 1, 2.5, 5, 10, 20, 50, 100, and 200 μmol/ml, 24 hr	↑ SOD activity ↓ NLRP3, NF-κB, IL-1β, cleaved-caspase1	(136)
*In vitro *plus* In vivo*				
*In vitro*, LS174T cells		5 μM, 24 hr	↑Expression of genes downstream of Nrf2	(139)
*In vivo*, Male C57BL/6 WT mice		200 mg/kg, 3 days, IG	↑Expression of genes downstream of Nrf2, activation of Nrf2 pathway ↓Colitis inflammation score, inflammatory markers, NLRP3 inflammasome	
*In vitro*, Caco-2 cells		10, 20, 40 μmol/l, 6 hr	↑ Sirt1 activity ↓ NLRP3, caspase-1, and ASC	(140)
*In vivo*, Male Wistar rats		20 mg/kg, IP	↓ASC-positive area in the intestinal epithelium, expressions of NLRP3 and caspase-1	
*In vivo*				
Male C57BL/6 J mice		8 mg/kg, 4 weeks, osmotic pump		↑Glucose control ↓Serum and liver triglyceride, hepatic levels of IL-1, IL-6 and TNF-α, expression of ASC and NLRP3	(19)
Male C57BL/6J mice		24 mg/kg, 6 months, PO		↓Hepatic IL-1β, COX-2, and TNF-α, mRNA level of NALP-3 inflammasome, ASC, caspase-1, NALP-1, and NALP-3	(142)
Male Sprague-Dawley rats		15 mg/kg, 5 days, IP		↓Intestinal injury, levels of IL-1β, IL-18, and TNF-α, mast cells and NLRP3 inflammasome activation	(143)
Male Sprague-Dawley rats		10 mg/kg, 20 weeks, PO		↓ Blood glucose, serum insulin, and triglyceride	(144)
Male C57/6 mice		50, 100, and 200 mg/kg, 21 days, gavage		↓Bowel inflammation, expression of NLRP3 and IL-1β	(145)
Female Shan-ma ducks		400 mg/kg, 15 days, PO		↑Villus height/crypt depth ratio, number of goblet cells, activation of Sirt1-NRF1/NRF2 signaling pathways, levels of ATP, SOD, and CAT ↓Jejunum histopathological damage, levels of NLRP3, caspase1 p20, and IL-1β	(146)
Male-specific pathogen-free ducks		500 mg/kg, 10 weeks, PO		- Regulated NF-κB/NLRP3 and Nrf2/Keap1 pathways ↑ Oxidative balance ↓ Aflatoxin B1-DNA adducts	(147)
Male Sprague-Dawley rats		50 mg/kg, 45 days, PO		↓NLRP3 inflammasome in the ileum and plasma, levels of IL-1α and IL-6 in ileum	(148)
Male C57BL/6J mice		500 mg/kg, 8 and 12 weeks, PO		↓Protein levels of NLRP3, ASC, caspase, IL-1β	(149)
Reproductive system				
*In vitro *				
HTR-8/SVneo cells		50 μmol/l, 8 hr	↑ Cell survival ↓ Oxidative stress, inflammasome activity	(152)
*In vitro *plus* in vivo*				
*In vitro*, Leydig and Sertoli cell lines		0-10 μM, 24 and 48 hr	↓ Inflammasome activation, endoplasmic reticulum stress, steroidogenic acute regulatory, CYP11A1, IL-1β, IL-18	(153)
*In vivo,* Male Sprague-Dawley rats		5 and 20 mg/kg, 6 weeks	- Preserved Sertoli and Leydig cell populations ↑ Epididymal and testicular weights, spermatogenesis, ↓ Histological abnormalities	
*In vivo*				
Female Sprague–Dawley rats		20 mg/kg, 30 days	↓ Metabolic and reproductive disturbances, including estrous cycle irregularities, obesity, ovulation disorders, serum testosterone, lipopolysaccharide levels, IL-6, TNF-α, NLRP3, pyroptosis, cleaved-caspase-1, GSDMD, IL-1β, IL-18	(154)
Male Wistar rats		20 mg/kg, 4 weeks, gavage	↑ LH, FSH, testosterone, sperm quality, CAT, SOD, expression of Ki-67 ↓ Testicular degeneration, MDA, caspase-3, NLRP3	(155)

ASC: apoptosis-associated speck-like protein containing a CARD domain; ATP: adenosine triphosphate; CAT: catalase; COX-2: cyclooxygenase-2; DNA: deoxyribonucleic acid; FSH: follicle-stimulating hormone; GSDMD: Gasdermin D; IL: interleukin; Keap1: Kelch-like ECH-associated protein 1; LH: luteinizing hormone; MDA: malondialdehyde; mRNA: messenger ribonucleic acid; NF-κB: nuclear factor-kappa B; NLRP3: nucleotide-binding domain, leucine-rich–containing family, pyrin domain–containing; Nrf: nuclear factor erythroid 2-related factor; SOD: superoxide dismutase; TNF-α: tumor necrosis factor-alpha

## Declaration

During the preparation of this work, the author(s) used ChatGPT in order to rephrase to reduce plagiarism and improve the language and grammar. After using this tool/service, the author(s) reviewed and edited the content as needed and take(s) full responsibility for the content of the publication.
